# Radiation-induced tumor immune microenvironments and potential targets for combination therapy

**DOI:** 10.1038/s41392-023-01462-z

**Published:** 2023-05-19

**Authors:** Siyu Guo, Yihan Yao, Yang Tang, Zengfeng Xin, Dang Wu, Chao Ni, Jian Huang, Qichun Wei, Ting Zhang

**Affiliations:** 1 Department of Radiation Oncology, Second Affiliated Hospital, Zhejiang University School of Medicine, Zhejiang University, Hangzhou, China; 2 Key Laboratory of Tumor Microenvironment and Immune Therapy of Zhejiang Province, Second Affiliated Hospital, Zhejiang University School of Medicine, Zhejiang University, Hangzhou, China; 3 Cancer Institute (Key Laboratory of Cancer Prevention and Intervention, National Ministry of Education), Second Affiliated Hospital, Zhejiang University School of Medicine, Zhejiang University, Hangzhou, China; 4 Cancer Center, Zhejiang University, Hangzhou, China; 5 Department of Orthopedic Surgery, Second Affiliated Hospital, Zhejiang University School of Medicine, Zhejiang University, Hangzhou, China; 6 Department of Breast Surgery, Second Affiliated Hospital, Zhejiang University School of Medicine, Zhejiang University, Hangzhou, China

**Keywords:** Drug development, Cancer microenvironment, Tumour immunology

## Abstract

As one of the four major means of cancer treatment including surgery, radiotherapy (RT), chemotherapy, immunotherapy, RT can be applied to various cancers as both a radical cancer treatment and an adjuvant treatment before or after surgery. Although RT is an important modality for cancer treatment, the consequential changes caused by RT in the tumor microenvironment (TME) have not yet been fully elucidated. RT-induced damage to cancer cells leads to different outcomes, such as survival, senescence, or death. During RT, alterations in signaling pathways result in changes in the local immune microenvironment. However, some immune cells are immunosuppressive or transform into immunosuppressive phenotypes under specific conditions, leading to the development of radioresistance. Patients who are radioresistant respond poorly to RT and may experience cancer progression. Given that the emergence of radioresistance is inevitable, new radiosensitization treatments are urgently needed. In this review, we discuss the changes in irradiated cancer cells and immune cells in the TME under different RT regimens and describe existing and potential molecules that could be targeted to improve the therapeutic effects of RT. Overall, this review highlights the possibilities of synergistic therapy by building on existing research.

## Introduction

Over half of cancer patients undergo radiation therapy (RT). RT can directly induce cancer cell death through various mechanisms, such as apoptosis, necrosis, and autophagy.^1^ Understanding the mechanisms of cellular changes post-RT to maximize radiation-related damage to cancer cells remains an ongoing goal. Furthermore, inflammatory mediators released by irradiated dying cells can attract and regulate immune cells in the tumor microenvironment (TME), further killing cancer cells. In addition, tumor-associated antigens (TAAs) produced by irradiated tumor cells can be captured by antigen-presenting cells (APCs) in the TME and presented to T cells. Activated T cells continue circulating throughout the whole system and sequentially function in unirradiated metastases. This phenomenon is called the abscopal effect and was previously rarely observed.^2,3^ However, some cancers, such as gastrointestinal adenocarcinoma and pancreatic cancer, are not sensitive to RT, resulting in poor efficacy and a high recurrence rate. Therefore, RT alone is not enough for cancer treatment, and combination therapy, including dual, triple, or even quadruple treatments, has been tested, showing excellent and long-lasting therapeutic results.

The emergence of immunotherapy has recently disrupted the paradigm of traditional cancer treatment (including the three traditional treatments RT, chemotherapy, and surgery), and immunotherapy functions by activating the body’s immune system to fight cancer. Immunotherapy mainly includes immune checkpoint inhibitors (ICIs), such as inhibitors of PD-1 (programmed cell death 1)/programmed cell death ligand 1 (PD-L1). Immunotherapy was shown to significantly increase the rate of the abscopal effect and prolong patient survival.^3^ However, less than 30% of patients benefit from immunotherapy.^4^ According to the number of tumor-infiltrating lymphocytes, tumors can be divided into three phenotypes: immune-inflamed, immune-excluded, and immune-desert types. Immune-inflamed tumors are called hot tumors, while the latter two are collectively referred to as cold tumors, and they respond poorly to immunotherapy.^5,6^ To a certain extent, RT can overcome the limitations of ICIs by turning cold tumors into hot tumors. Thus, RT combined with ICIs represents a new treatment model. A phase II study showed that patients with resected local-regionally advanced head and neck squamous cell carcinoma (HNSCC) had improved survival when pembrolizumab was added to adjuvant RT (NCT02641093).^7^ However, the sequence of administration of ICIs and RT remains debatable. One study reported an increased survival benefit with simultaneous RT compared with sequential administration,^8^ while another study found no significant difference between the two strategies,^9^ possibly because simultaneous RT plus immunotherapy and the administration of an ICI before RT may kill cancer cells as well a substantial number of immune cells, leading to poor systemic response and toxic side effects.^10^ Additionally, it was found that the synergistic effects were weakened when the period between RT and immunotherapy was too far apart.^8,11^ However, clinical trials suggest that a sufficient interval between immunotherapy and RT might be necessary to reduce serious side effects associated with combination therapy.^12^ Thus, balancing the toxicity and efficacy of combination therapy remains a challenge.

RT is a double-edged sword that can cure cancer patients but also damage normal tissues. Irreversible damage is caused when the radiation dose exceeds the repair capacity of the surrounding normal tissues. Hence, improving the accuracy of RT and reducing the radiation dose without impairing the efficacy of RT are the main goals of current research. The former can be achieved by applying three-dimensional conformal radiotherapy (3D-CRT) and conformal intensity-modulated radiotherapy (IMRT) techniques. Presently, it is possible to protect surrounding healthy tissues while maximizing the radical RT dose to the tumor. Comparatively, the latter requires extensive research into the tumor immune microenvironment (TIME) to understand the interactions between cells in the TIME after RT to find more RT sensitizers related to immune cells.

In this review, we summarize how cancer cells and different immune cells in the TME respond to RT and discuss the potential immune cell-related RT sensitizers that can be used to enhance the effects of RT (Table 1).

**Table 1:** Current and potential targets of radiotherapy sensitization

Type of cells	Target	Drug	Mechanisms of drugs	Ref
Cancer Cell	DNA	Bi NPs Gold NPs [AuNPs] Phy@PLGdH nanosheets Hafnium oxide NPs [NBTXR3] X-ray (AGuIX) nanoparticles	Increasing DNA Damage	^26,48,49^
Cancer Cell	Trex	-	Inhibiting DNA degradation	^38,41^
Cancer Cell	Caspase 8	zVAD	Promoting necroptosis	^40^
Cancer Cell	SLC7A11	Erastin Sulfasalazine	Promoting ferroptosis	^222^
Cancer Cell	GPX4	RSL3 ML162	Promoting ferroptosis	^222^
Cancer Cell	ATG4B	NSC185058	Inhibiting autophagy	^223^
Cancer Cell	Autophagosome	Chloroquine	Inhibiting autophagy	^224^
Cancer Cell	PARP	Niraparib	Inhibiting DDR Promoting T cell infiltration	^28,29^
Cancer Cell	DNA-PK	AZD7648	Inhibiting DDR	^30,31^
Cancer Cell	ATM	AZD1390 XRD-0394	Inhibiting DDR	^35^
Cancer Cell	ATR	AZD6738 BAY 1895344	Inhibiting DDR	^34^
Cancer Cell	Wee1	ZN-C3 AZD1775 IMP-7068	Inhibiting DDR	^32,33^
Cancer Cell	CHK1/2	AZD7762	Inhibiting DDR	^36^
Cancer Cell	PI3K	GDC-0084 XL765 BKM120 BYL719 BR101801	Inhibiting DDR	^54,55^
Cancer Cell	mTOR	NVP-BEZ235	Inhibiting DDR	^56^
Cancer Cell	MEK	U0126 AZD6244 Trametinib GSK2118436B	Inhibiting DDR	^24^
Cancer Cell	TAA	Antigen-capturing stapled liposome (ACSL)	Inhibiting TAA degradation	^51^
Cancer Cell	TAA	Salmonella (coated with antigen-adsorbing cationic polymer NPs)	Promoting TAA aggregation	^50^
Cancer Cell DC	STING DNGR-1 (CLEC9A)	PLGA/STING@EPBM nanovaccines	Deliverying’ 2’,3’-cGAMP Antigen presentation	^43^
Cancer Cell, DC, TAM, MDSC, T cell, Breg	STING	Alg-Mn^2^^+^ NaGdF4:Nd@NaLuF4@PEG-polyphenol/Mn (DSPM) TMA-NP NP-cGAMP cGAMP/MOL	Type I IFN preduction DC activation M1 polarization Inhibting CD8 + T cell terminal differentiation	^44–46,176,177^
Cancer Cell	CD47	Hu5F9-G4 CC-90002 IBI188	Promoting phagocytosis	^25,116^
Cancer Cell	FAO	Etomoxir	Inhibiting CD47	^58^
Cancer Cell	VEGF	Bevacizumab Avastin	Inhibiting tumor vessel growth	^107^
Cancer Cell	TGF-α	TNFerade™ Tianenfu Enbrel^R^	Inhibiting tumor vessel growth	^107^
DC	TLR4	ANPs rBCG-S.FimH PA-MSHA APS LBPL MPLA GLA-SE	Promoting DC activation M1-phenotype polarization	^87^
DC, TAM	TLR7/8	R848 CDNP NKTR-262 cN@SS-IMQ NIA-D1@R848 The Smac-TLR7/8 hydrogel	Promoting DC activation M1-phenotype polarization	^89^
DC, TAM, B cell	CD40	Sotigalimab(APX005M) PVA-CD40 2141-V11 LVGN7409 CDX-1140	Promoting DC maturation	^189^
DC	TLR9	CpG SD101 T-nanoCpG CpG@Au NPs	Promoting DC activation M1-phenotype polarization	^88,120^
DC	YTHDF1	–	Promoting Antigen presentation	^225^
DC	Tagln2	Recombinant dU-TG2P	Promoting Antigen presentation	^226^
DC	SHP1	Vitamin E SSG	Promoting Antigen presentation	^227^
DC	sGSN	–	Promoting Antigen presentation	^70^
DC	Non-canonical NF-κB signaling	–	Promoting type I IFN production	^73^
DC	MDA5/LGP2	HMW Poly I:C	Promoting DC enrichment	^78^
TAM, MDSC	CCL2-CCR2	MC21 INCB3344	TAM recruitment MDSC recruitment	^99,132^
TAM	CCL2-CCR2 CCL5-CCR5	BMS-687681	TAM recruitment	^102^
TAM, MDSC, TAN	CSF1-CSFR	GW2580 PXL3397	TAM/MDSC proliferation, differentiation and migration TAN recruitment N1-phenotype polarization	^101,130^
TAM	WNT3A	–	Inhibiting Cancer Cell mesenchymal transition	^98^
TAM	sICAM-1	–	TAM recruitment Inhibiting WNT3A production	^98^
TAM, MDSC	Arg1	Nor-NOHA ADI-PEG20	Inhibiting M2-phenotype M1-phenotype polarization	^104,142^
TAM	HMGB1	–	M1-phenotype polarization	^96^
TAM	Mertk	UNC2025	Efferocytosis M1-phenotype polarization	^112^
TAM, MDSC	IDO	INCB023843 Indoximod BMS-986205	Inhibiting M2-phenotype	^141^
MDSC	ROCK2	–	M1-phenotype polarization	^106^
TAM	FGF2	–	M1-phenotype polarization	^109^
Cancer Cell TAM, MDSC, TAN, Treg	TGF-β	SM16 Fresolimumab SHR 1701	M1-phenotype polarization TAN recruitment N1-phenotype polarization	^111,148^
TAM	BACE1	MK-8931	M1-phenotype polarization	^90^
DC, TAM	SIRPα	TTI-621 ALX148	Promoting phagocytosis Promoting Antigen presentation M1-phenotype polarization	^115,116^
Cancer Cell TAM	DNA TAM	ZGd NRs	Increasing DNA Damage Depleting TAM	^118^
Cancer Cell TAM	Mannose Levamisole hydrochloride	M/LM-Lipo	Promoting glycolysis Promoting PD-L1 degradation Inhibiting autophagy	^125^
MDSC	Lactate	Hf-MOLs	Reducing hypoxia	^136^
MDSC	TGF-β PD-L1	Bintrafusp alfa SHR-1701	Inhibiting immunosuppressive effects	^139^
MDSC	PDE5	Sildenafil	Decreasing MDSC	^142^
MDSC	ATRA	L-ATRA	Promoting MDSC differentiation	^143^
MDSC	PERK NRF2	–	Reprogramming MDSCs	^133^
TAN	IFN-β	–	N1-phenotype polarization	^147^
TAN	PAD4	–	Inhibiting NETosis	^150^
TAN	NETs	DNAse I Elastase inhibitor (NEi)	Degrading NETs	^150^
TAN	Glut1	–	Enhancing neutrophils’ turnover	^156^
Eosinophil	IL-5	Recombinant mouse IL-5	Promoting eosinophil expansion	^158^
Immune Cells	PD-1	Sintilimab Camrelizumab Camrelizumab Pembrolizumab SHR-1210 Nivolumab Tislelizumab TSR-042	Reversing immune cell function	^60,76,161,183,187,208^
Cancer Cell	PD-L1	Durvalumab Atezolizumab Avelumab	Reversing immune cell function	^161^
DC, T cell	PD-L1 B7-1&2	PD-L1xCD3ε Durvalumab	Promoting DC activation Reversing immune cell function	^228^
Treg	CTLA4	Ipilimumab BMS-986218 Tremelimumab	Reversing immune cell function	^161^
Immune Cells	TIM-3	Anti-TIM-3	Reversing immune cell function	^74–76,161,178^
Immune Cells	LAG-3	Anti-LAG-3	Reversing immune cell function	^161^
T cell, NK cell	TIGIT	Anti-TIGIT	Reversing immune cell function	^161,187,188^
Myeloid cells, Naive T cell	VISTA	Anti-VISTA	Reversing immune cell function	^161^
T cell	STAT3	CpG-STAT3ASO STAT3 ASO	Reducing Treg	^170,171^
T cell, NK cell	IL-2	NKTR-214 BEMPEG	Promoting CTL and NK cell activation and proliferation	^173–175^
T cell, NK cell	CD25	Anti-CD25	Deleting Tregs Promoting CTL and NK cell activation and proliferation	^168,178,212^
T cell	OX40	α-OX40	Promoting CD8^+^ T cell activation	^183^
T cell	GITR	α-GITR	Promoting CD8^+^ T cell activation	^182,184^
NK cell, T cell	4-1BB	α-4-1BB	Promoting NK cell activation Promoting CD8^+^ T cell activation	^186,201^
T cell	ICOS	α-ICOS	Promoting CD8^+^ T cell activation	^185^
NK cell	Histone deacetylase	HDACi	Amplifying NK cell cytotoxicity	^203^
NK cell	Membrane-bound TRAIL Soluble TRAIL (sTRAIL)	IFN-β	Amplifying NK cell cytotoxicity	^207^

## RT-induced signaling pathways in cancer cells

DNA damage is caused directly by ionization or indirectly by reactive oxygen species (ROS) produced by RT. At this point, cells have been exposed to radiation in sublethal doses and initiated procedures to restore cellular homeostasis, such as the DNA damage response (DDR), autophagy, and the unfolded protein response (UPR).^1^ In cases in which the cellular damage is irreparable, the cell may enter a state of cellular senescence or undergo processes related to immunogenic cell death (ICD),^1^ such as necroptosis, ferroptosis and pyroptosis.^13,14^ Senescent cancer cells can still survive but permanently stop growing and cannot reenter the cell cycle, which seems to be beneficial to antitumor effects. However, they do not stop secreting immunosuppressive cytokines, such as transforming growth factor (TGF)-β1 (which is also secreted by senescent cancer-associated fibroblasts (CAFs)) and chemokine (C-X-C motif) ligand 2 (CCL2), known as the senescence-associated secretory phenotype (SASP); this cytokine secretion attracts myeloid cells with immunosuppressive phenotypes, including myeloid-derived suppressor cells (MDSCs) and M2-like tumor-associated macrophages (TAMs).^1,15^ A previous study reported that eliminating senescent cells using ABT-263 can attenuate glioblastoma (GBM) growth and improve the therapeutic effect of RT.^16^

RT results in single-strand breaks (SSBs) and double-strand breaks (DSBs) related to dose as well as linear energy transfer (LET).^1^ LET ranges from 0.3–1 keV/μm (X and γ rays) to 10 keV/μm (protons), 10–100 keV/μm (carbon ions), 116 keV/μm (α particles) or 270 keV/μm (^36^Ar ions).^17,18^ The following three types of particle therapies are currently used for cancer treatment: proton beam therapy (PBT), carbon-ion beam therapy (CIBT), and boron neutron capture therapy (BNCT).^19^ High-LET RT is highly lethal because, due to a large amount of energy deposition, it causes DNA cluster damage and changes in chromatin structure that are more severe and difficult to fix.^20^ In addition, a Bragg peak is formed when proton rays reach the tumor.^19^ In other words, the energy ray is fully released only when reaching the tumor site without affecting healthy tissues around the tumor.^21^ Studies found that there was no significant difference in the amount of high mobility group protein B1 (HMGB1) between X-ray-irradiated TMEs and carbon ion-irradiated TMEs,^22^ but the bystander effect produced by photon RT was stronger than that of carbon ion RT, demonstrating that carbon ion RT may produce fewer side effects.^23^ However, the use of high-LET RT is limited in the clinic and causes different changes in cancer cells and subsequently in the TIME. Comparatively, low-LET RT induces single DNA damage, such as DSBs, most of which can be repaired, leading to changes in tumor cell signaling, which causes further changes in the TIME. Thus, in this review article, we only review the effects of photon RT on the tumor immune microenvironment.

## DNA damage repair-related signal pathways

DNA damage caused by RT includes single-strand breaks (SSBs) and double-strand breaks (DSBs). Poly (ADP-ribose) polymerase (PARP) plays an important role in the repair of SSBs. DSBs are widely repaired by nonhomologous end joining (NHEJ), a process that requires DNA-dependent protein kinase (DNA-PK) involvement in the early stage, mediated by the RAS–RAF–MEK–ERK pathway.^24^ In addition to NHEJ, high-fidelity homologous recombination repair (HRR) is also important in the repair of DSB where DNA-PK is also involved. DNA damage can lead to stepwise phosphorylation of the ataxia-telangiectasia mutated (ATM)-checkpoint kinase 2 (CHK2)-p53 pathway. p53 activation, together with phosphorylation of the RAD3-related (ATR)-CHK1-cyclin-dependent kinase 2 (CDK2) and WEE1-CDK1 pathways, also contributes to inducing reversible cell cycle arrest.^24^ Especially when the TP53 gene is mutated, the G1 phase blockade becomes dysfunctional, making the cells completely dependent on the G2/M checkpoint for the DDR. The ATR/CHK1 pathway upregulates PD-L1 and CD47 and is related to the activation of STAT3 transcription.^25^ Therefore, inhibiting the G2/M checkpoint has been recognized as important to radiosensitize TP53-mutant tumors.

The radiosensitivity of cancer cells can be increased by increasing DNA damage. Nanoparticle (NP)-carried chemotherapeutic drugs have already been proven to be effective in clinical practice.^26^ For instance, a dual-target NP using both a cisplatin prodrug and a histone deacetylase inhibitor increased DNA damage, impaired cancer cell repair ability, and promoted the effects of RT.^27^ The radiosensitivity of cancer cells can also be increased by inhibiting the DDR. The mechanism of PARP inhibitors, such as niraparib, is that PARP forms cross-links with proteins during DNA damage, triggering the collapse of replication forks, resulting in the accumulation of DSBs in cells in S phase and their dependence on HRR.^28,29^ AZD7648, a potent and specific inhibitor of DNA-PK, plus RT was shown to induce complete tumor regression in MC38 and CT26 models, which was dependent on the presence of CD8^+^ T cells rather than natural killer (NK) cells.^30,31^ Inhibition of Wee1 by ZN-C3, AZD1775, or IMP-7068 can lead to G2 checkpoint abolition, allowing unrepaired DNA to persist into the mitosis phase, triggering mitotic catastrophe and eventually cell death.^32,33^ Greater tumor regression can be observed when the ATR inhibitor AZD6738 is added to RT.^34^ The ATM inhibitor (AZD1390) plus RT is being investigated in a phase I clinical trial in brain cancer (NCT03423628).^35^ The inhibition of CHK1/2 by AZD7762 can abolish G2/M phase cell cycle arrest and promote the progression of mitosis to produce more micronuclei after RT.^36^

## The cGAS-STING signaling pathway

Damaged DNA fragments and micronuclei accumulated in the cytosol of irradiated cancer cells can act as damage-associated molecular patterns (DAMPs), which can be sensed by cyclic GMP-AMP synthase (cGAS) to produce cGAMP. Then, cGAMP activates stimulator of interferon genes (STING) to produce type I interferon (IFN), which promotes dendritic cell (DC) maturation or binds to IFNR on cancer cells in an autocrine manner.^37,38^ The formation of micronuclei attaches to cell cycle progression through mitosis after DBS.^39^ At the same time, tumor-derived exosomes released after RT (RT-TEX) containing cytosolic DNA can activate the cGAS-STING pathway.^38^ In addition to mitochondrial instability, the release of mitochondrial DNA (mtDNA) after the activation of necroptosis mediated by the Z-DNA binding protein 1 (ZBP1)-receptor-interacting protein kinase 3 (RIPK3)-mixed lineage kinase domain-like (MLKL) pathway can also activate cGAS-STING signaling. Caspase 8 acts as a converter to cause cancer cells undergoing necroptosis to shift to undergo apoptosis, and only cells undergoing necroptosis can produce type I IFN, which is essential for maintaining the inflammatory irradiated TME and activating the subsequent immune response.^40^ The DNA exonuclease Trex1 produced by cancer cells after high-dose radiotherapy (HDRT, 12–18 Gy) can degrade cytosolic DNA in both cancer cells and RT-TEX, indicating that Trex1 can lead to less activation of DCs, thereby reducing the effects of RT. Thus, the administration of STING agonists and inhibition of Trex1 could be attractive methods to increase tumor immunogenicity.^38,41^ Amplification of the STING signaling cascade can activate multiple signaling pathways, such as the NF-κB and interferon regulatory factor (IRF3) pathways, to achieve the complex effects of STING signaling; these are mainly immune-activating effects, such as DC activation, M1 polarization, inhibiting the immunosuppressive effects of MDSCs and inhibiting the terminal differentiation of CD8^+^ T cells. However, the use of STING agonists enhanced antitumor immunity in mouse models, proving that the pathway has a much greater impact on immunostimulatory cells than other immunosuppressive cells. Therefore, STING agonists have been valued for their sensitizing effects in tumor therapy and have shown promising results in mouse models. However, the clinical effect has not been ideal, which might be related to the infiltration of MDSCs, STING amplification of Bregs and the promotion of interleukin (IL)-35 secretion by downstream activation of IRF3. In addition, the regulation of PD-L1 by ATR/CHK1 and STING/TANK binding kinase 1 (TBK1)/IRF3 could be another potential reason for the nonideal effects.^42^

The cGAS-STING pathway can be directly targeted. For instance, RT combined with the engineered PLGA/STING@EPBM nanovaccines targeting DNGR-1 to deliver the STING agonist 2′,3′-cGAMP, and this strategy was found to be more effective than TAAs. The administration of Mn^2^^+^ within 24 h after RT was shown to increase the binding affinity of cGAS for cytosolic DNA.^43^ Mn^2^^+^ carried by alginate (Alg-Mn^2^^+^), NaGdF4:Nd@NaLuF4@PEG-polyphenol/Mn (DSPM), and TMA-NP containing the STING agonist c-di-AMP can induce continuous release of Mn^2^^+^, and this was shown to have synergetic effects with RT in CT26, B16, and 4T1 mouse models.^44–46^

The cGAS-STING pathway can also be indirectly activated by increasing TAAs. By exacerbating RT-induced DNA damage to induce potent in situ tumor vaccination and generating more ROS in cancer cells, activation and guiding of irradiation by X-ray (AGuIX) NP, Phy@PLGdH nanosheets (a hybrid nanoplatform [MGTe]) and metal NPs (i.e., Gold NPs [AuNPs], Hafnium oxide NPs [NBTXR3] and Bi NPs) can produce more TAAs and have already shown synergetic effects with RT in B16, CT26, and 4T1 mouse models.^26,47–49^ Even more interestingly, intratumoral injection of Salmonella coated with antigen-adsorbing cationic polymer NPs was shown to promote DC activation by gathering TAAs around the tumor in the CT26 model, and these TAAs could be easily degraded in lysosomes.^50^ An antigen-capturing stapled liposome (ACSL) design was shown to capture and transport TAAs from lysosomes to the cytoplasm of DCs, enhancing the effects of RT by promoting TAA cross-presentation in a 4T1 mouse model.^51^

## The PI3K/AKT signaling pathway

**Fig. 1:**
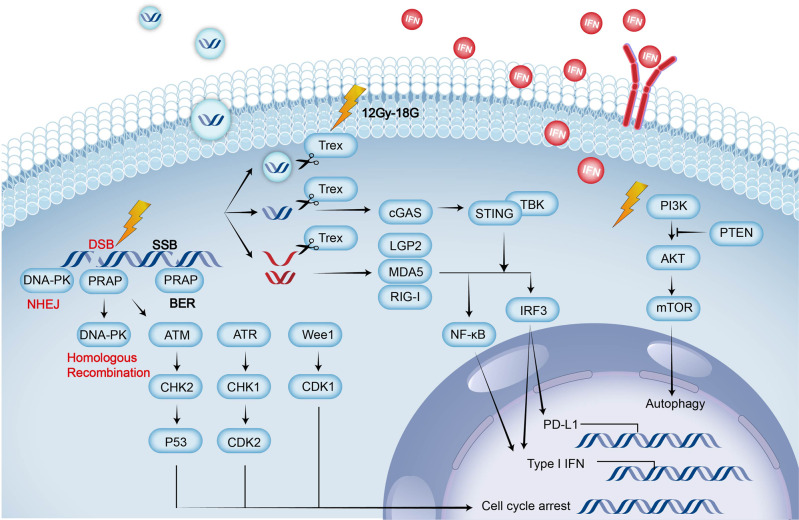
Radiation on pathways of cancer cells. Radiation causes DNA damage, which leads to cell cycle arrest via the ATM/CHK2/p53 pathway, ATR/CHK1/CDK2 pathway, and Wee1/CDK1 pathway. SSB and DSB are the two main forms of DNA damage, and PARP is involved in repairing both of them. SSBs are mainly repaired by BER, while DSBs are mainly repaired by NHEJ and HRR related to DNA-PK. The cytosolic DNA and RNA generated after RT act on downstream NF-κB and IRF3 by activating the cGAS-STING pathway and RLRs (including LGP2/MDA5/RIG-1), respectively, leading to upregulation of type I IFN and PD-L1. Type I IFN can act on other cells or cancer cells in an autocrine manner. At the same time, this cytosolic DNA can also be transported extracellularly by extracellular vesicles to function outside the cells. The DNA and RNA can be degraded by Trex produced by HDRT (12–18 Gy). The PI3K/AKT pathway can also be upregulated after RT to regulate autophagy. RLRs, RIG-I-like receptors; BER, base excision repair; NHEJ, nonhomologous DNA end joining; HRR, high-fidelity homologous recombination repair

Overactivation of the phosphatidylinositol 3-kinase (PI3K)/AKT pathway after RT is closely related to tumor radioresistance. Activation of NF-ΚB and mTOR, which are downstream of AKT, promotes cell survival by enhancing the DDR and mediating autophagy and apoptosis.^24,52^ The PI3K-AKT pathway plays a key role in maintaining the transcriptional translation of hypoxia inducible factor-1α (HIF-1α) in tumors.^53^ Inhibiting the PI3K-AKT-mTOR pathway can reduce tumor hypoxia and induce arrest at the G2/M phase in cancer cells sensitive to DNA damage by RT. Class I PI3K molecules include a catalytic subunit (α, β, γ, δ). Both PI3Kαδ and PI3Kγδ inhibitors can enhance the synergetic effects of RT and anti-PD-L1 therapy in CT26 and triple-negative breast cancer (TNBC) mouse models.^54,55^ The mTOR kinase inhibitor NVP-BEZ235 can slow the DDR by inducing the phosphorylation of DNA-PK to significantly increase the radiosensitivity of HNSCC cells.^56^ HER2 can activate the PI3K-AKT pathway and continuously activate the NF-κB pathway to promote CD47 gene transcription. The expression of CD47 on cancer cells acts as a “don’t eat me” signal to prevent cancer cells from being phagocytosed because CD47 binds signal regulatory protein α (SIRPα) on APCs, including DCs and macrophages. In HER2-expressing breast cancer cells, radioresistance occurs when CD47 and HER2 are upregulated following RT via the HER2-NF-κB pathway. It was found that RT combined with dual antibodies had a better effect than RT combined with a single-target antibody because of the crosstalk between HER2 and CD47.^57^ In addition to glycolysis, mitochondrial fatty acid oxidation (FAO) also provides cellular fuel to cancer cells by burning saturated fat. FAO-derived acetyl-CoA acetylates NF-κB/RelA K310 to upregulate CD47 transcription and prevent GBM cells phagocytosis by macrophages. Inhibiting FAO with etomoxir can greatly affect radioresistant GBM cells and improve the effects of RT.^58^ (Fig. 1).

## Dendritic cells

Different types of DCs function differently. Conventional type I dendritic cells (CD103^+^cDC1) cross-presented antigens on MHC class I to CD8^+^ T cells, recruited T cells to the tumor by producing CXCL9 and CXCL10 and produced IL-12 after sensing IFN-γ released from T cells,^59,60^ leading to the initiation and maintenance of antitumor immunity and increasing antitumor immunity in a mouse model, while CD11b^+^ cDC2s specifically primed CD4^+^ T cells and were heterogeneous.^61,62^ mregDCs are a recently discovered subtype of DCs. They are derived from DC1s and DC2s, and their differentiation is associated with the uptake of tumor antigens by DC1s and DC2s.^63^ It was found that the PGE2-EP2/EP4 pathway can activate NF-κB gene transcription to promote mregDCs to recruit regulatory T cells (Tregs), but the mechanism by which mregDCs change after RT still needs to be elucidated.^64^

In the draining lymph nodes (TdLNs) of an MC38 model, a radioimmunogenic tumor-bearing mouse model, RT did not change the number of tumor-migratory cDCs but promoted the maturation of DCs by upregulating the expression of the costimulatory molecules CD40 and CD80. Migration occurred when mature DCs expressing CCR7 bound CCL7 released by cancer cells after RT. In the Pan02-SIY tumor model, the number of DCs was decreased in poorly radioimmunogenic tumors, and the phenotype did not change, indicating that RT is ineffective in some tumors and the urgent need for exogenous adjuvants as RT sensitizers.^62^

After RT, dying tumor cells can act as ‘vaccines in situ’ when they release DAMPs, such as calreticulin (CRT), ATP, HMGB1, F-actin, dsDNA and cytosolic RNA. Both autophagy and the UPR are essential in this process. The UPR can lead to exposure of CRT on the plasma membrane of dying cells. By binding to LRP1 (known as CD91), CRT acts as an ‘eat me’ signal to enhance phagocytosis of dying cells.^65^ Autophagy can produce ATP by degrading cytoplasmic materials, and successful autophagy can keep cancer cells alive. However, initiated but ultimately failed autophagy can cause cell death and release substantial ATP. ATP acts as a ‘find me’ signal to recruit DCs via the P2Y2 receptor on their surface. ATP can also activate NLRP3 inflammasomes via the receptor P2X7 on DCs to produce IL-1β and promote DC cross-priming by binding to IL-1R1 on DCs.^1,66^ RT upregulates CD39 via the STAT1-IRF1 axis. CD39 can hydrolyze ATP to produce immunosuppressive adenosine. CD39 blockade can cause the accumulation of ATP and promote ICD induction by RT.^67^ Enhanced ICD was shown to increase the therapeutic effects of CAR-T cells in mouse GBM models. In addition, HMGB1 not only had a synergistic effect with ATP to stimulate NLRP3 inflammasomes but also prevented antigens from being degraded by controlling the fusion of phagosomes and lysosomes via Toll-like receptor 4 (TLR4) to promote antigen presentation.^68,69^ As mentioned above, DAMPs binding to pattern recognition receptors (PRRs) on the surface of DCs promotes processing and presentation of TAAs by DCs (particularly cDC1s). Dendritic cell natural killer lectin group receptor-1 (DNGR-1, also known as CLEC9A) on cDC1s can enhance the presentation of antigens by binding to F-actin exposed on dead cells. The effects of DNGR-1 can be antagonized when plasma protein-secreted gelsolin (sGSN) competes with DNGR-1 by binding with F-actin. Therefore, blocking sGSN could be a potential strategy for enhancing the effects of RT.^70^ Activated DCs migrate to the draining lymph nodes to present antigens to naïve T cells, leading to an increase in CD8^+^ T cells.^71^ It was previously believed that the central nervous system had no lymphatic vessels. However, the existence of meningeal lymphatic vessels (MLVs) was shown to contribute to the trafficking of DCs. VEGF-C was reported to induce MLV expansion, and injection of VEGF-C mRNA significantly enhanced RT efficacy in a GL261 GBM mouse model.^72^

Cytosolic DNA acts via the cGAS-STING pathway inside DCs. The cGAS-STING pathway can activate canonical NF-κB signaling, which has a dual role in the TME. On the one hand, the canonical NF-κB pathway is essential for the antitumor response because it supports type I IFN secretion by DCs in the TME after local RT; thus, inhibiting the NF-κB pathway may dampen the effects of RT. On the other hand, the canonical NF-κB pathway might cause tumor radioresistance by inducing the expression of antiapoptotic proteins such as BCL2 apoptosis regulator and caspase 8 (CASP8-) and FADD-like apoptosis regulator (CFLAR, also known as c-FLIP).^1^ In contrast, the function of noncanonical NF-κB signaling is much simpler.^73^ It can also be activated by cGAS-STING signaling and block the generation of type I IFN by inhibiting the binding of RelA to the *ifnb* promoter in DCs.^73^ These results indicate that specifically inhibiting noncanonical NF-κB signaling may increase the curative effects of RT.

Extracellular DNA uptake and the activation of inflammasomes are restrained by T-cell immunoglobulin and mucin-containing molecule 3 (TIM-3) on DCs, suggesting a novel mechanism for TIM-3 inhibitors.^74,75^ After specifically knocking out TIM-3 in DCs, tumor growth was significantly inhibited in mouse models, and this effect was stronger than that of knocking out TIM-3 in T cells.^74^ The synergistic effects of RT+anti-TIM-3 have not been ideal. Studies reported that in mice depleted of CD4^+^ T cells or CD8^+^ T cells, no or only a marginal difference was observed between mice treated with anti-TIM-3 alone versus anti-TIM-3+RT. There have been no studies to verify whether removing TIM-3 from DCs can significantly enhance the effects of combining with RT, and this may be a promising future direction.^76^

In addition, it was reported that RT can also produce cytosolic RNA, which can be sensed by PRRs, including RIG-I-like receptors (RLRs). RLRs are composed of RIG-I, melanoma differentiation-associated protein 5 (MDA5), and laboratory of genetics and physiology 2 (LGP2).^77^ The gene expression of LGP2 and MDA5 has been found to be associated with DC enrichment.^78^ LGP2 was reported to be linked with CD8^+^ T cell survival in antiviral responses^79^; later, LGP2 was found to be essential for the antitumor effects of RT because LGP2-deficient BMDCs cannot cross-prime T cells since IFNβ induction in response to irradiated tumor cells is greatly impaired. Thus, high molecular weight (HMW) dsRNA Poly I:C treatment, a synthetic MDA5/LGP2 agonist, might be a promising to improve the therapeutic effect of RT.^78^

Bacillus Calmette-Guérin (BCG) instillations and RT are used for urothelial carcinoma in clinical practice. BCG can not only cause the release of the cytokines IL-1, IL-2, TNF, and IFN-γ by urothelial cells but also increase the number of CD4^+^ T cells and the expression of tumor necrosis factor-related apoptosis-inducing ligand (TRAIL) on both neutrophils and Th1 cells in the TME.^80,81^ A study reported that BCG, but not 15 Gy RT, generated an immune response in urothelial carcinoma via the activation of the cGAS-STING pathway due to a high level of Trex produced by HDRT.^82^ Accordingly, whether the combination of low-dose radiotherapy (LDRT) and BCG can produce better effects remains to be further investigated.

After intratumoral administration of DCs with RT, 9 of 17 (52.9%) patients with soft-tissue sarcoma developed a tumor-specific immune response, and there was a remarkable abscopal effect in castration-resistant prostate cancer patients.^83,84^ LDRT (4 Gy) plus the TLR9 agonist SD-101 led to tumor shrinkage in all patients with indolent lymphoma and nonirradiated tumor shrinkage in 24 of 29 patients.^85^

**Fig. 2:**
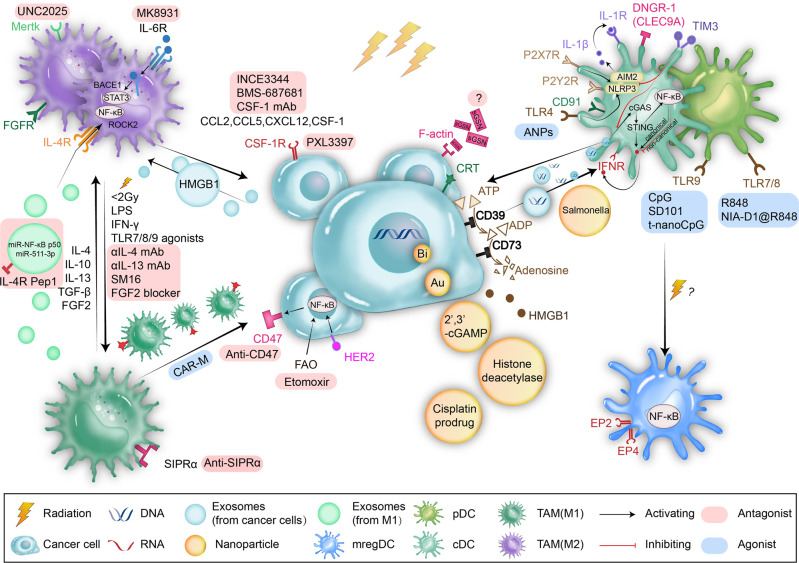
Radiation on DCs and TAMs. DAMPs derived from irradiated cancer cells, such as CRT, ATP, HMGB1, and F-actin, can promote DC processing and presentation of TAAs by binding the receptors CD91, P2Y2/P2X7, TLR4, and DNGR-1, respectively, on DCs. dsDNA accumulated after radiation in the cytoplasm or exosomes activates the cGAS-STING pathway, resulting in the production of type I IFN, which is an important factor promoting DC maturation. Targeting the cGAS-STING pathway or targeting receptors that sense DAMPs by locally injecting agonists or injecting agonists wrapped around NPs can activate DCs and further promote the effects of radiotherapy. Irradiated cancer cells attract TAMs into the TME by releasing CCL2, CCL5, CXCL12, and CSF-1. Inhibiting these chemokines with INCE3344, BMS-687681, and CSF-1 mAb may prevent TAMs (mostly of the immunosuppressive M2 phenotype) from infiltrating the TME and enhance the therapeutic effects of RT. IL-4, IL-10, IL-13, TGF-β, FGF2 and HMGB1 carried in tumor exosomes can shift immune-stimulating M1-like macrophages into M2-like macrophages. Either promoting M1 polarization by LDRT (<2 Gy), LPS, IFN-γ and TLR agonists, or preventing M2 polarization by SM16, FGF2 blockers, anti-IL-4 mAb or anti-IL-13 mAb shows synergetic effects with RT. Another strategy to stop M2 polarization is to modify exosomes released by M1 macrophages with IL-4R Pep1 to target IL-4R expressed on M2-like macrophages and induce the release of NF-κB p50 miRNA and miR-511-3p to target ROCK2 and NF-κB p50 at the same time. DC, dendritic cell; CRT, calreticulin; HMGB1, high mobility group protein B1; ATP, adenosine triphosphate; TLR, Toll-like receptor; IFN, interferon

Compared with DCs or TLR agonists directly administered into the tumor for radiation sensitization, NPs targeting DCs represent a more effective antigen delivery system; such strategies are termed NP vaccines, and they have shown better treatment efficacy and less toxicity.^43,86,87^ Bioactive polysaccharide NPs (ANPs) can not only promote DC maturation by regulating the NF-κB pathway via TLR (particularly TLR4) and upregulate costimulatory molecules such as CD40, CD80, and CD86 but also enhance the abscopal effect of RT by reshaping the immunosuppressive TME.^87^ Compared with the TLR9 agonist CpG, t-NanoCpG administered intravenously and intranasally can better penetrate the blood‒brain barrier (BBB) and strongly stimulate the maturation of dendritic cells to treat glioma. The combination of t-NanoCpG with RT further improved the survival rate of LCPN glioma-bearing mice.^88^ Intravenous administration of the TLR7/8 agonist R848 plus RT reversed the immunosuppressive TME of PDAC and inhibited both primary PDAC and hepatic metastatic tumor growth.^89^ Furthermore, a more functional three-in-one NP called NIA-D1@R848 including R848, the RT sensitizer NIA and the PD-L1 antagonist D1 was designed and demonstrated a more significant therapeutic effect when combined with RT^89^ (Fig. 2).

## Tumor-associated macrophages

Approximately half of tumor-infiltrating immune cells are TAMs, which consist of a small number of tumor-suppressive TAMs (sTAMs) and a large number of tumor-promoting TAMs (pTAMs). Classically activated (M1) macrophages express markers such as inducible NO synthase (iNOS), CD86, and IL-12, while alternatively activated (M2) macrophages express high levels of IL-10, arginase 1 (Arg1), and CD206. sTAMs exhibiting M1-like macrophage phenotypes are thought to have an antitumor effect, while pTAMs exhibiting M2-like macrophage phenotypes are considered tumor-promoting.^90^ The polarization of M1-like macrophages requires stimuli such as IFN-γ produced by NK cells and Th1 cells, or TLR ligands such as lipopolysaccharide (LPS). These macrophages produce proinflammatory cytokines such as TNF-α. Additionally, cytokines such as IL-4, IL-13, and IL-10 produced by Th2 cells can drive macrophages into an M2-like phenotype, enabling them to produce anti-inflammatory cytokines such as TGF-β and IL-10 via the JAK/STAT signaling pathway.^91^

Different doses of radiation may have different indirect effects on TAM polarization. Radiation, especially high single-fraction RT doses, can create a hypoxic TME, in which tumor cells produce CCL2, CCL5 and CXCL12 to recruit macrophages to create an immunosuppressive TME.^1,92^ Colony stimulating factor-1 (CSF-1), which is abundant in the hypoxic TME, contributes to the proliferation, differentiation and migration of TAMs by binding CSF-1R on TAMs.^93^ RT can drive the Th2 polarization of CD4^+^ T cells, causing them to express the cytokines mentioned above to promote M2 polarization. In addition, tumor-derived exosomes released during RT-mediated immunogenic cell death can also increase M2 polarization by increasing glycolysis conversion, resulting in upregulation of PD-L1.^94^ M2-like macrophages remain radioresistant regardless of oxygen concentration.^95^

Intriguingly, it was shown that LDRT (2 Gy) promoted M1 repolarization, reduced PD-1 expression and enhanced TAM phagocytosis.^1,96^ Although repolarization can be mediated by exosomes containing a higher level of HMGB1 produced by irradiated cells, the underlying mechanism is still unclear.^96,97^ Soluble intercellular adhesion molecule-1 (sICAM-1) produced by GBM cells after RT contributes to the infiltration of macrophages. sICAM can cause macrophages to produce WNT3A, which transforms GBM cells into a mesenchymal phenotype by binding leukocyte function-associated antigen 1 (LFA-1) on TAMs. Thus, WNT3A or sICAM could be potential targets to increase sensitivity to RT.^98^

On the one hand, one of the main strategies to overcome radioresistance and improve the therapeutic effects of RT is limiting the infiltration of macrophages in the TME. Inhibiting the CCL2-CCR2 interaction with INCB3344 can reduce the amount of M2 macrophages recruited into both MB49 bladder tumors and pulmonary metastases after RT.^99^ Administration of a monoclonal antibody (mAb) targeting CSF-1 or targeting CSF-1R with an inhibitor (PXL3397) was confirmed to enhance the effect of RT by reducing macrophage infiltration. Tumor growth was delayed in mammary carcinoma mouse models,^100^ although in phase I (NCT02452424) and a phase II trial (NCT03336216), PXL3397 plus anti-PD-L1 therapy failed to exhibit satisfying therapeutic effects.^101^ Administration of the dual inhibitor BMS-687681, targeting both CCR2 and CCR5, led to a superior survival rate when combined with RT+αPD-1 therapy in a PDAC mouse model.^102^

On the other hand, another way to address radioresistance could be by inducing M1 polarization of M2 macrophages. STING agonists are useful for achieving M1 polarization, especially in BRCA1-deficient breast cancer, which features more M2 polarization.^103^ ADI-PEG20, an inhibitor of Arg1, was shown to be effective in an arginosuccinate synthetase 1 (ASS1)-negative GBM tumor model. However, due to the absence of CpG island methylation, ASS1-positive GBM cells were unaffected by ADI-PEG20. Surprisingly, complete tumor elimination was observed when RT was combined with ADI-PEG20. The combination promoted the recruitment and M1 repolarization of macrophages/microglia.^104^ Compared with M1-polarized macrophages, M2-polarized macrophages exhibit a higher level of IL-4R. M2 polarization is required for NF-κB p50. Knockout of NF-κB p50 in mice is associated with increased RT responses.^105^ IL-4Rα is a receptor for both IL-4 and IL-13, and neutralizing either with αIL-4 mAb or αIL-13 mAb can control the regrowth of tumors after RT.^100^ After using IL-4RPep-1 to modify the surface of M1 exosomes to target IL-4R on M2-like macrophages, M1 macrophage exosomes transfected with NF-κB p50 miRNA and miR-511-3p were found to be internalized into M2 macrophages. Thus, separately knocking down NF-κB p50 expression and targeting rho-associated coiled-coil containing protein kinase 2 (ROCK2) promotes M2 polarization of macrophages by phosphorylating the transcription factor IRF4, leading to the repolarization of M2 macrophages into M1 macrophages.^106^ However, more research is needed to clarify the synergistic effect between modified M1 macrophage exosomes and RT.

TNF-α, an antitumor cytokine at high concentrations, can also promote tumor growth at low concentrations. TNF-α production by macrophages can be upregulated after RT. Radiation resistance occurs when TNF-α directly mediates the differentiation of monocytes into angiogenic cells, which support tumor vessel growth, and promotes VEGF production by macrophages by binding with TNFR on macrophages in an autocrine manner. VEGF produced by macrophages can induce tumor vessel abnormalities and hypoxia, making cells resistant to RT. Targeting either TNF-α with Enbrel ^®^ or VEGF can increase radiosensitivity.^107^

After irradiation, the phagocytic receptor Mer tyrosine kinase (MerTK), which is specific to macrophages and not found on DCs, and the MerTK ligands (Gas6 and Pros1) on apoptotic cells are upregulated. Exposed phosphatidylserine (PS) on apoptotic cells can bind Gas6 and then MerTK-Gas-PS activity can activate macrophages to phagocytose apoptotic cells. This apoptotic cell-clearance process is also called efferocytosis.^108^ Efferocytosis, TGF-β and fibroblast growth factor 2 (FGF2), an angiogenic growth factor discovered before VEGF, can all contribute to M2-like macrophage polarization.^108–110^ Compared with RT alone, RT combined with an FGF2 blocker can effectively delay MC38 tumor growth.^109^ Pretreatment with the TGF-β receptor I antagonist SM16 before RT improved the efficacy of RT in a CT26 tumor model, but the Panc02 tumor model responded poorly.^111^ Combination of the MerTK inhibitor UNC2025 with RT prolonged the survival of GBM mouse models.^112^ In the context of loss of MerTK and TGF-β signaling, RT can be curative even in the Panc02 tumor model.^113^ When TAMs were depleted by clodronate liposomes, the efficacy of a large single dose of RT (20 Gy) or fractionated dose of RT (2×20 Gy) was improved, but the effects of TGF-βR blockade were abrogated in head, neck, and lung cancer models.^107,114^

BACE1 is a transmembrane aspartyl protease expressed by pTAMs. It can cleave IL-6R to produce sIL-6R, following which the sIL-6R/IL-6 complex activates STAT3. pTAM maintenance depends on the trans-IL-6/sIL-6R/STAT3 pathway. Inhibition of BACE1 by MK-8931 repolarized pTAMs into sTAMs by reducing STAT3 activation in a GBM model. Considering the low TAM infiltration in GBM, it is not surprising that there was also a synergetic effect when MK-8931 was combined with LDRT(2 × 2 Gy), which markedly increased TAM infiltration.^90^

When CD47 binds to SIRPα on macrophages, it can promote the clearance of tumor cells. Bian et al. reported that adoptive transfer of SIRPα^−/−^ macrophages, but not anti-CD47 treatment, with RT resulted in durable tumor regression because the macrophages could reverse an the immunosuppressive TME by expressing the M1 phenotype. Although both methods can block the CD47-SIRPα axis, targeting SIRPα can increase responses to RT because CD47-independent pathways are triggered following SIRPα activation.^115^ M1 macrophage exosomes modified with anti-CD47 and -SIRPα antibodies can abolish the “don’t eat me” signal and promote M1 macrophage repolarization at the same time.^116^ When CD47 binds to SIRPα on DCs, it can promote TAA uptake and presentation to T cells. RT+anti-CD47/anti-SIRPα+anti-PD-1 antibodies triple therapy was found to induce vigorous systemic antitumor immunity based on the induction of TAA-specific CD8^+^ T cell priming by DCs and the initiation of CD8^+^ T cell expansion and activation.^25^ More interestingly, studies have shown that only under the premise of DAMPs released by cancer cells after RT, SIRPα-deficient macrophages can obtain a strong ability to present TAAs to T cells.^25,115^

Similar to CAR-NK cell therapy, CAR-M therapy has recently emerged as a novel approach, and relevant studies are currently in the early stages. Such therapy may overcome two main limitations of CAR-T cell therapy: immune cell infiltration and an immunosuppressive TME. Encouragingly, macrophages are abundant in the TME, and changing their phenotypes can reverse the immunosuppressive TME. Due to these unique advantages, CAR-M therapy may be a promising radiosensitizer.^117^

Nanoimmunotherapy aims to reduce the number of TAMs or drive M1 repolarization. ZGd NRs can deposit X-rays to induce ICD and deplete TAMs simultaneously. A remarkable synergetic effect has already been demonstrated in both 4T1 breast cancer metastasis and CT26 models.^118^ CDNPs loaded with TLR-7/8 agonist, Smac-TLR7/8 hydrogel, CpG@Au NPs with the TLR9 agonist CpGs and a multifunctional NP composed of polylysine, iron oxide and CpGs (PIC) can effectively promote M2 macrophage repolarization into M1 macrophages after RT.^92,119,120^ In addition, combination of PIC+RT with α-PD-1 therapy led to tumor regression in GBM mouse models.^121^ Combining a PD-L1 inhibitor with LDRT (2 Gy) plus Bi-nMOF led to significant tumor regression accompanied by an increased proportion of M1 macrophages in cold tumor models, such as the TRAMP-C2 and Panc02 tumor models.^122^

Interfering with the metabolic processes of cancer or immune cells is a new research direction. TAMs rely on glycolysis to maintain their rapid proliferation, similar to cancer cells. Mannose restrains both TAMs and cancer cells by inhibiting glycolysis and PD-L1 degradation.^123,124^ Levamisole hydrochloride can recruit lysosomes in TAMs. Excessive lysosomes can augment mannose’s effects to inhibit glycolysis by degrading the M6P isomerase enzyme and increase the autophagy of cancer cells and TAMs.^124^ A biocompatible liposome loaded with mannose and levamisole hydrochloride (M/LM-Lipo) achieved a prominent therapeutic effect when combined with RT in 4T1 mouse models because the strategy decreased the numbers of both cancer cells and immunosuppressive M2 macrophages by interfering with cell metabolism.^125^ (Fig. 2).

## Myeloid-derived suppressor cells

In normal circumstances, bone marrow-derived myeloid cells differentiate into macrophages, DCs and granulocytes. However, in pathological circumstances, they differentiate into MDSCs.^126^ MDSCs are a mixture of polymorphonuclear myeloid-derived suppressor cells (PMN-MDSCs) and monocytic cells (M-MDSCs). They are an important group that maintains the tumor immunosuppressive microenvironment by expressing Arg1, iNOS, ROS, TGF-β, indoleamine-2,3-dioxygenase 1 (IDO), and PD-L1 to inhibit T cell proliferation.^37,123,127^ PMN-MDSCs are immature neutrophils, and it is difficult to distinguish PMN-MDSCs from the protumor phenotype neutrophils (N2s) because both are identified as CD11b^+^LY6G^+^LY6C^low^ cells in mice. M-MDSCs are immature mononuclear cells that have the potential to differentiate into TAMs.^128^ However, in humans, LOX1 expression can distinguish PMN-MDSCs from neutrophils, while the lack of HLA-DR expression can distinguish M-MDSCs from monocytes.^129^

Radiation can cause tumor cells to release chemokines and growth factors, such as CCL2, CCL7, and CSF-1, that are important for MDSC recruitment in the TME. The nonreceptor tyrosine kinase ABL1 is activated by radiation before it translocates to the promoter region of CSF-1 and promotes the expression of CSF-1 in cancer cells.^130^ MiR-26b-5p in EVs derived from dying tumor cells after irradiation can also contribute to the activation of MDSCs via the PTEN/PI3K/AKT pathway.^131^ DNA damage caused by RT activates both the MDSC-intrinsic and MDSC-extrinsic cGAS-STING pathways to produce more IFNβ, which stimulates cancer cells to produce the CCR2 ligands CCL2 and CCL7.^132,133^ After RT, MDSCs infiltrate the stroma of tumors by activating the CCL2/CCL7-CCR2 and CSF-1-CSF-1R axes.

Different RT regimens may lead to dynamic changes in MDSC recruitment. The level of MDSCs in the tumor is low throughout fractionated radiation (5×3 Gy) treatment. The level begins to increase from the third day after RT and then decreases after peaking on the sixth day.^130^ However, treatment with a high single radiation dose (15 Gy or 30 Gy) significantly decreased the number of MDSCs by 14 days after a brief increase in a colon cancer mouse model, but this pattern not observed with fractionated radiation (3 × 10 Gy) treatment. We hypothesized that this might be because of the high infiltration levels of T cells caused by the high single radiation dose, which resulted in the production of high levels of cytokines, such as TNF-α, IFN-γ and FasL, that can directly kill MDSCs.^126^ Radiation induces a hypoxic TME by upregulating the expression of glycolytic enzymes, such as LDHA, PKM2 and HIF-1α, in PDAC, resulting in enhanced glycolysis and increased lactate production. The activity of MDSCs is promoted via the GPR81/mTOR/HIF-1α/STAT3 pathway when lactate binds to its receptor GPR81 on MDSCs.^134^ In addition, HIF-1α can upregulate PD-L1 on MDSCs by binding to the PD-L1 promoter, which causes radioresistance by restraining the effect of CD8 + T cells.^135^

Aggregation of MDSCs in the TME after RT is one of the main causes of radioresistance. After inhibiting CCL2/CCR2 with the anti-CCR2 antibody MC21 or the CSF-1/CSF-1R pathway with the inhibitor GW2580, the influx of MDSCs after RT is directly inhibited.^130,132^ RT plus a STING agonist can trigger a robust antitumor effect but simultaneously activate the cGAS/STING pathway to induce MDSC accumulation. Thus, the administration of MC21 can further enhance STING agonist plus RT combination treatment by eliminating MDSCs.^132^ Targeting lactate by inhibiting LDHA or administering Hf-MOLs to deliver ROS to achieve successful RT-RDT with LDRT are safe strategies that indirectly reduce the number of MDSCs by regulating the TME.^136^ Combining Hf-MOL-enabled RT-RDT with anti-PD-L1 therapy not only eradicated the local tumor but also limited lung metastasis in a 4T1 model by significantly reducing both subtypes of MDSCs observed in the primary tumor as well as the lung.^137^ Combining RT with anti-PD-L1 therapy significantly reduced the number of MDSCs because cytokines produced by T cells, especially TNF, can directly lead to the apoptosis of MDSCs after anti-PD-L1 restores T-cell function.^53,138^ Bintrafusp alfa (BA) and SHR-1701 are bifunctional fusion proteins that can simultaneously inhibit TGF-β and PD-L1.^139^ In 4T1 tumor-bearing mice, the BA+RT (BART) strategy significantly reduced the numbers of the two subtypes of MDSCs in the tumor. By lowering the level of plasma granulocyte colony-stimulating factor (G-CSF), BART also restrained tumor metastasis because G-CSF causes PMN-MDSCs to accumulate in the lungs, leading to the formation of the lung premetastatic niche.^140^ The median survival of the 4T1 mouse model was further increased when the mice were treated with BART plus M3814, which suppressed the repair of DSBs.^30^

Weakening the immunosuppressive effects of MDSCs in the TME can also counteract radioresistance. For instance, inhibiting Arg1 with nor-NOHA or IDO with INCB023843 after RT effectively increased the number of CD8^+^ T cells and delayed tumor growth.^141,142^ The PDE5 inhibitor sildenafil was reported to reduce the proportion of PMN-MDSCs and the expression of Arg1 in MDSCs in the Lewis lung carcinoma (LLC) mouse model, indicating that a combination of sildenafil and RT might be a promising strategy to overcome radioresistance.^142^

**Fig. 3:**
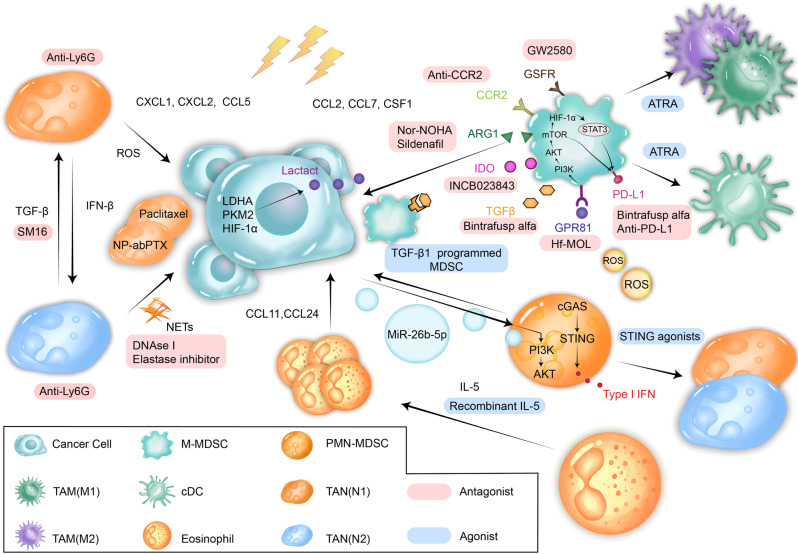
Effects of radiation on MDSCs, TANs, and eosinophils. Infiltration of MDSCs is one of the most important causes of radiation resistance. CCL2, CCL7, and CSF-1 released after RT attract MDSCs into the TME by binding CCR2 and CSFR. Radiation-induced hypoxia can upregulate the expression of PD-L1 on MDSCs and cause cancer cells to produce lactate, which activates MDSCs by binding GPR81. Hf-MOL reduces lactate by carrying ROS to improve the hypoxic state of the TME. Extracellular vesicles from irradiated cancer cells encapsulating miR-26b-5p activate MDSCs via the PI3K/AKT pathway. STING agonists inhibit the immunosuppressive effects of MDSCs by activating cGAS-STING signaling in MDSCs. TGF-β-MDSCs cultured in TGF-β1-conditioned media were found to be immuno-activating and to have less expression of PD-L1 and more expression of FasL; thus, they can strongly increase the effects of RT. Blocking the inflow of MDSCs with anti-CCR2 antibodies and GW2580 or reducing the production of immunosuppressive factors, such as Arg1, IDO, and TGF-β, with bintrafusp, nor-NOHA, sildenafil or INCB023843 can prevent the immunosuppressive effects of MDSCs. Another way to reduce the number of MDSCs in the TME is to use ATRA to promote MDSC maturation into DCs and TAMs. Radiation activates neutrophils to release granule proteins and NETs to promote cancer cell metastasis. Depleting neutrophils with anti-Ly6G therapy and eliminating NETs with DNAse I or elastase inhibitors can reduce the negative effects of TANs. SM16 targets TGF-β, a factor causing immunosuppressive N2 phenotype polarization. Trials of SM16 or other TGF-β inhibitor combinations that aim to sensitize cells to RT are ongoing. In addition, RT-recruited neutrophils can function as an excellent delivery system to transport drugs, such as albumin-bound paclitaxel, to cancer cells. CCL11 and CCL24 are important chemokines that attract eosinophils into the TME, and IL-5 is a vital cytokine contributing to eosinophil proliferation. Administering recombinant IL-5 can amplify the antitumor effects of eosinophils. MDSCs, myeloid-derived suppressor cells; Arg1, arginase 1; IDO, indoleamine 2,3-dioxygenase; ATRA, all-trans retinoic acid; NETs, neutrophil extracellular traps; ROS, reactive oxygen species

In addition, changing the phenotype of MDSCs represents a novel strategy to inhibit the immunosuppressive effects of MDSCs. Trans-retinoic acid (ATRA) can promote MDSC differentiation into mature myeloid cells.^127^ Administration of a engineered liposome containing actively loaded ATRA (L-ATRA) lowered tumor growth and promoted M-MDSC differentiation into DCs in a CT26 colorectal mouse model.^143^ RA improved the therapeutic efficacy of RT in B16, CT26 and renal carcinoma models. RT+RA combination treatment further enhanced the abscopal effects of anti-PD-L1 therapy, although the synergetic effects of the dual and triple treatments were mostly from the recruitment and differentiation of inflammatory macrophages (inf-MACs).^144^ TGF-β-MDSCs are stimulatory MDSCs derived from conditioned medium in the presence of TGF-β1. Surprisingly, TGF-β-MDSCs cultured by human MDSCs also have the same immuno-activating properties as murine TGF-β-MDSCs. The inhibitory effects on T cells were abrogated by downregulating the expression of iNOS in murine TGF-β-MDSCs and PD-L1 in human TGF-β-MDSCs. The upregulation of FAS-L directly led to the death of tumor cells in vitro and in vivo. TGF-β-MDSCs seem to be a promising radioenhancer because RT combined with intratumoral administration of TGF-β-MDSCs can result in the regression and long-term tumor control of cancerous lesions in vivo.^127^ Ablative the PKR-like ER kinase (PERK) or the nuclear factor-erythroid-2-related factor 2 (NRF2), which is downstream of PERK in MDSCs induce the release of mtDNA as a result of oxidative stress and reprogram MDSCs into immunostimulatory cells by activating the intrinsic cGAS-STING pathway. Compared with PERK-expressing MDSCs, PERK-deficient tumor-derived MDSCs can more efficiently process and present antigens. However, further investigations are needed to understand the synergistic effect with RT^133^ (Fig. 3).

TAMs and MDSCs have many overlapping features; for example, they share chemokines (CCL2), growth factors (CSF-1), and immunosuppressive effectors (such as Arg1, IDO, and TGF-β). Therefore, many agents can jointly target TAMs and MDSCs; however, some targets are also unique to each cell type, such as MerTK and WNT3A (Table 1).

## Tumor-associated neutrophils

The term tumor-associated neutrophils (TANs) specifically refers to blood neutrophils attracted into the TME not only by CXCL1, CXCL2, CXCL6, and CXCL8 expressed by tumor cells but also by IL-17 expressed by γδT cells.^145^ Similar to TAMs, TANs can be divided into two phenotypes: the antitumor phenotype (N1) and the N2.^146^ The conversion between N1 and N2 depends on cytokines in the TME. For instance, the presence of IFN-β promoted TAN polarization into the N1 phenotype, while TGF-β induced the opposite effects.^147,148^ Activated neutrophils release granule proteins to activate the Notch pathway signaling in lung alveolar type II cells and then enhance breast cancer metastasis to the lungs.^149^ Activated neutrophils release neutrophil extracellular traps (NETs) through a process called NETosis, and it was shown that neutrophils could be recruited into radiated and nonradiated TMEs in the tumor-bearing bladder, while NETs could only be detected in irradiated TMEs.^150^ In the bloodstream, NETs can capture and transfer circulating tumor cells, contributing to tumor metastasis.^129,151^ In the TME, NETs can block CD8^+^ T cell infiltration into tumors as a barrier, and a high polymorphonuclear neutrophil (PMN)-to-CD8^+^ T cell ratio was shown to be related to poor survival in a bladder cancer model.^150^

RT can induce sterile inflammation, characterized by the infiltration of neutrophils.^152^ Radiation to healthy lung tissues creates a pro-metastatic microenvironment by increasing neutrophil infiltration and promoting neutrophil degranulation.^149^ Comparatively, radiation to tumor cells causes persistent DNA damage, whereby dying cancer cells release chemoattractants such as CXCL1, CXCL2, and CCL5 to recruit neutrophils. TANs were increased at 12 h due to RT-mediated infiltration after a slight decrease at 6 h, which might be related to direct killing by radiation, and subsequently, the neutrophil number peaked within 24 h.^152,153^ Furthermore, RT can also lead to the production of HMGB1, which can promote NETosis by binding TLR4 on neutrophils.^150^ However, as the existing studies on TANs after radiation are limited and controversial, more studies are needed for clarification.

Neutrophils promote resistance to high-dose (20 Gy) radiation, and depleting TANs with anti-Ly6G antibody in combination with RT delays tumor growth by downregulating MAPK pathway signaling in locally advanced cervical cancer driven by the MAPK pathway.^154^ In contrast, it was found that a combination of 5 Gy RT and depletion of neutrophils did not delay tumor growth in an FC1242 PDAC model characterized by a high level of TANs.^155^ Thus, the role of TANs in the TME after radiation is still controversial. Therefore, much effort is still needed to explore the effects of different doses and fractionations of RT on TANs in different models. Knocking out PAD4 (an important enzyme in mice during NETosis), degrading NETs with DNAse I or administering an elastase inhibitor (NEi) can overcome radiation resistance by increasing the infiltration of CD8^+^ T cells.^150^

Glut1 is a glucose transporter involved in glycolysis. TANs express more Glut1 and have greater preferences for glycolysis than neutrophils in healthy lungs, which prolongs neutrophil survival. Thus, the depletion of Glut1 can improve the effects of RT by shortening the lifespan of TANs and enhancing neutrophil turnover, indicating that younger neutrophils have fewer opportunities to acquire markers such as PD-1 than older neutrophils and can attack tumor cells to overcome RT resistance.^156^ This trait makes RT-recruited neutrophils (RT-Ns) a useful tool for delivering drugs such as albumin-bound paclitaxel. Chen et al. found that local RT activated neutrophils, causing them to engulf albumin NPs (NP-abPTX), and led them to the tumor. This drug transport strategy was associated with fewer side effects and demonstrated synergetic effects with RT.^157^

Takeshima et al. found that RT-Ns produced more ROS than neutrophils originally residing in tumors, and ROS resulted in oxidative damage and apoptosis of cancer cells, which enhanced the therapeutic effects of RT.^152^ Administrating G-CSF after irradiation marginally increased the number of RT-Ns, the production of ROS by RT-Ns, and the upregulation of N1 markers such as ICAM1 and TGF-α.^152,153^ Inhibition of TGF-β by SM16 remarkably increases the expression of neutrophil chemoattractants such as CXCL2, CXCL5, and CCL3 and simultaneously promotes N1 polarization, although the antitumor effect is mostly dependent on T cells.^148^ Therefore, modulating neutrophil polarization at a specific stage could be a promising strategy to precisely target N2 neutrophils and preserve N1 neutrophils. It may also have a synergetic effect with RT and effectively hinder the development of tumors^129^ (Fig. 3).

## Eosinophils

Eosinophils contribute to the recruitment and antitumoral effect of CD8^+^ T cells, which are attracted by CCL11 and CCL24 produced by cancer cells after irradiation. When eosinophils are depleted, signatures such as those related to the recruitment and activation of T cells are reduced. Eosinophils are an integral part of the antitumor immune response, and their abundance is related to better progression-free survival (PFS) in patients with non-small cell lung cancer (NSCLC) and nasopharyngeal carcinoma (NPC) after RT. Improved tumor control can be observed when RT is combined with recombinant mouse IL-5 to increase the number of eosinophils, indicating that expanding eosinophils could be a potential radiosensitization strategy^158^ (Fig. 3).

## T cells

T cells, actually αβT cells, can be divided into CD8^+^ T cells and CD4^+^ T cells. Naïve CD4^+^ T cells can differentiate into T helper (Th) cells, including Th1, Th2, Th9, and Th17 cells and CD4^+^ Tregs. CD4^+^CD25^+^FoxP3^+^ Tregs can suppress effector T cells by secreting immunosuppressive cytokines, such as TGF-β, and producing adenosine, and this is amplified when Tregs undergo apoptosis.^159^ In addition, Tregs can also impair the function of APCs by binding to the coinhibitory receptor cytotoxic T lymphocyte antigen 4 (CTLA-4) via two mechanisms. One mechanism is by binding CD80/B7-1 and CD86/B7-2 on APCs, such as DCs and macrophages, because the affinity of the CTLA-4 for the ligands (CD80 and CD86) is higher than that of the costimulatory receptor CD28 expressed on cytotoxic T cells (CTLs) for CD80 and CD86. Another mechanism is that CTLA-4 can capture those ligands from APCs, especially from migratory DCs, by a process called transendocytosis.^160^ CD8^+^ T cells include CTLs, memory T cells (Tems) and CD8^+^ Tregs. Both CTLs and Th cells are effector T cells (Teff). Constant stimulation of antigens can cause Teff exhaustion, accompanied by the expression of inhibitory receptors such as PD-1, TIM-3, T cell immunoreceptor with immunoglobulin and ITIM domains (TIGIT), V-domain immunoglobulin suppressor of T cell activation (VISTA), and lymphocyte activation gene-3 (LAG-3). M1 macrophages with high expression of CD80, CD86 and MHCII can continuously stimulate Teff via TCR-MHCII binding, inducing T cell exhaustion.^161^ Exhausted T cells (Tex) can be divided into stem cell-like exhausted PD-1^+^CD8^+^ T cells and terminally exhausted PD-1^+^CD8^+^ T cells. Stem cell-like T cells are known for their self-renewal and expansion abilities, which contribute to long-lasting antitumor immunity.^162^

γδT cells, distinct from αβT cells, can detect changes during the transformation of healthy cells into cancer cells to monitor cancer development and eliminate them in time.^163^ With the development of a tumor, γδT cells in the tumor change from a PD-1^–^IFNγ^+^ antitumor phenotype to a PD^–^1^+^IL17^+^ IFNγ^−^ protumor phenotype.^164^ Exosomes secreted by γδT cells (γδ-T-Exos) can upregulate the expression of CCR5 on T cells to promote T cell migration. In addition, γδ-T-Exos can also induce the death of tumor cells via the FAS-FASL and death receptor 5 (DR5)/TRAIL axes.^165^

Radiation, especially HDRT, can directly influence the survival of CD8^+^ T cells. However, RT can cause DCs to present antigens to CD8^+^ T cells (as mentioned above), and IL-1 signaling in DCs leads to antigen presentation that ensures that T cells that have been exposed to HDRT can survive.^66^ In addition, RT can also induce the secretion of CXCL9, CXCL10, CXCL11, and CXCL16 to attract new CD8^+^ T cells into the TME.^166^ RT plus PARP inhibitors can activate the cGAS/STING signaling pathway and promote the production of cytokines, such as IFN-β, CCL5, and CXCL10, which is conducive to T cell recruitment.^167^ Compared with CIM, a combination of the RACIM regimen (cyclophosphamide+αPD-1 and αCTLA-4 blocking Ab+agonistic αCD40 Ab) with LDRT (<2 Gy) induced recruitment of both CD4^+^ T cells and CD8^+^ T cells, especially CD4^+^ T cells with a Th1 signature. Pseudotime analysis showed that the transition of Th1 cells into CD4^+^ Tpex and CD4^+^ Ttex in mice with LLC or ovarian cancer was a dynamic process similar to that of CD8^+^ T cells, suggesting that RACIM can effectively reverse this transition. The infiltration phenomenon has also been reported in a phase I clinical trial.^6^ Comparatively, the effects of Tregs have been found to be complicated. In an MC38 mouse model, a single dose of 12 Gy was found to increase the proportion of Tregs, which was decreased with 8 Gy.^168^ Similarly, in a B16 mouse model, researchers found that a single dose of 15 Gy increased the proportion of Tregs, while doses of 7.5 Gy and 10 Gy decreased it.^169^ In addition, low-dose fractionated RT (5 × 2.3 Gy) did not increase Tregs.^168^ Radiation also produces type I IFN and activates the NF-κB pathway via the cGAS-STING pathway, leading to IL-10 production. The binding of IL-10 to IL-10R activates STAT3 signaling, contributing to Treg proliferation, differentiation and immunosuppressive effects, including the expression of CTLA-4.^123^

Thus, the principle of combination therapy is to deplete Tregs and allow the proper functioning of Teffs.

Inhibition of STAT3 with CpG-STAT3ASO or STAT3 antisense oligonucleotides (ASOs) can reduce the proportion of Tregs in irradiated HNSCC and PDAC tumors in mouse models.^170,171^ IL-2 contributes to the activation and proliferation of Tregs, CTLs and NK cells, as previously described.^172^ FoxP3 induces the expression of CD25, a high-affinity receptor for IL-2, suggesting that Tregs may express more CD25 than CTLs and NK cells. Therefore, Tregs may better inhibit the functions and proliferation of T cells and NK cells by preferentially using IL-2.^159,168^ NKTR-214 is a recombinant IL-2 that is more likely to bind CD122 than CD25, resulting in enhancement of CTL and NK cell function. NKTR-214 + RT resulted in better survival of CT26 mice by reducing the Treg proportion and effectively promoting Teff infiltration and function in both irradiated and abscopal areas.^173^ Furthermore, RT+NKTR-214+NKTR-262 (a novel TLR7/8 agonist) generated more functional CD8^+^ T cells with less PD-1 expression in a CT26 mouse model.^174^ A similar phenomenon was observed when MC38 mice were treated with RT+anti-CD25 antibody.^168^ Bempegaldesleukin (BEMPEG) is an investigational CD122-preferential IL-2 pathway agonist. The synergetic effects of RT plus BEMPEG were observed among B78 melanoma, 4T1 and MOC2 mouse models.^175^

The stem-like phenotype of CD8^+^ T cells is maintained by increased TCF1 expression derived from the intrinsic cGAS-STING pathway. It was shown that STING agonists could improve CAR-T therapy by preventing CD8^+^ T cell terminal differentiation.^162^ Using different types of STING agonists, such as administrating the NP cGAMP/MOL and inhaling NP-cGAMP, was found to have synergetic therapeutic effects with RT; triple therapy using RT+a STING agonist+CAR-T was reported to exhibit strong therapeutic potential.^176,177^

The synergetic effects of RT combined with anti-PD-L1 therapy in activating T cells were found to be effective but transient.^53,178,179^ The combination reversed terminal exhaustion of PD−1^+^CD8^+^ T cells, causing them to transition into stem cell-like exhausted PD−1^+^CD8^+^ T cells and increased the expression of TIM-3 on CD8^+^ T cells and Tregs.^10^ A dual immune checkpoint blockade (αPD-L1+αTIM-3) reduced the infiltration of Tregs, but the effect faded with time, with Tregs reappearing. The use of anti-CD25 antibody to deplete Tregs and RT+αPD-L1+αTIM-3 demonstrated a durable and robust immune response.^178^ EVs loaded with siPDL1 can traverse the BBB and effectively increase the number of T cells in GBM. The synergetic effects of RT combined with administration of these EVs were verified in a recent study.^180^ However, LLC is resistant to RT+anti-PD-L1 therapy, and Olivo Pimentel et al. found that the immunocytokine L19-IL-2 performed better than anti-PD-L1 antibodies in the LLC mouse model.^181^ In addition to inhibiting coinhibitory receptors, stimulating costimulatory receptors, such as OX40, GITR, 4-1BB and ICOS, could also be a strategy to restore the antitumor ability of T cells and convert the Treg cells that accumulate after RT or ICI therapy into effector T cells.^182^ Since T cell exhaustion is a major cause of resistance to drugs that stimulate costimulatory receptors, the addition of anti-PD-1 antibodies may overcome this resistance. The RT+PD−1/CTLA-4 blockade+α-OX40/α-GITR/α-ICOS/α-4-1BB triple regimen achieved substantial improvement of primary tumors, metastasis control and improved survival outcomes with fewer exhausted T cells and Tregs in 4T1, NSCLC, Panc02, and GBM mouse models.^183–186^

The RadScopal technique refers to the application of LDRT to a metastatic area and the application of HDRT to the primary tumor. LDRT reduces the expression of PVR, the ligand of TIGIT, on APCs. Although there are no clinical trials on RT+anti-TIGIT therapy, combining RadScopal (HDRT: 3 × 12 Gy; LDRT: 2 × 1 Gy), anti-TIGIT therapy and anti-PD-1 therapy significantly affected outcomes by controlling both primary and metastatic 344SQ-P tumors.^187^ Adding FlT3L to increase CD103^+^ DC proliferation in the tumor site improved the synergetic effects of RT+anti-TIGIT therapy in MC38 and B16F10 mouse models.^188^

**Fig. 4:**
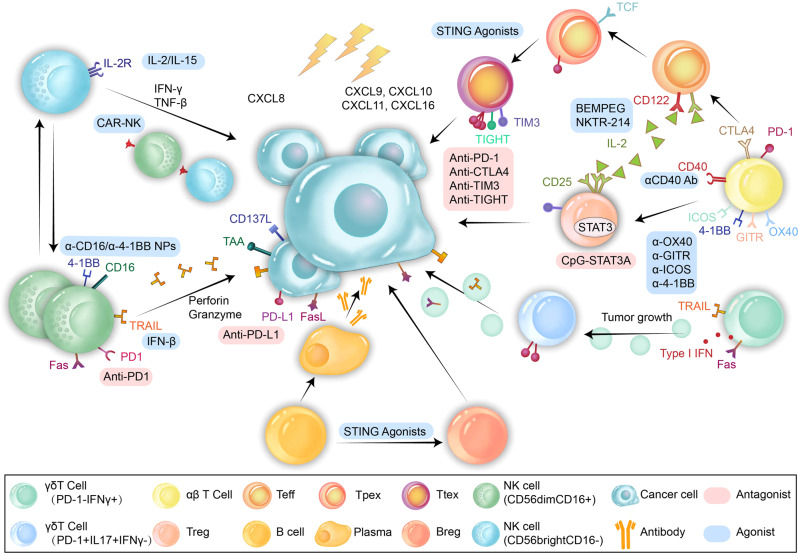
Effects of radiation on T cells, NK cells, and B cells. Radiation attracts both Teff and Tregs into the TME. Teff are the main cells responsible for cancer cell death but shift to the exhausted phenotype after RT. CD122 on Teff shares the same ligand with CD25 on Treg: IL-2. Selective activation of Teff by NKTR-214 or BEMPEG and removal of Tregs by CpG-STAT3ASO or anti-CD25 treatment can effectively sensitize cells to the therapeutic effects of RT. Stimulating the costimulatory receptors OX40/GITR/ICOS/4-1BB with α-OX40/α-GITR/α-ICOS/α-4-1BB treatment or inhibiting the coinhibitory receptors PD-1/CTLA-4/TIM-3/TIGIT can effectively prolong the action time of RT, exhibiting long-lasting effects of T cells. TRAIL and Fas receptors on γδT cells or in γδ-T-exos can effectively kill cancer cells. However, as the tumor grows, γδT cells shift to an immunosuppressive phenotype. Irradiated cancer cells activate the mTOR and NF-κB pathways, leading to the release of CXCL8, which attracts more CD56^dim^ NK cells than CD56^bright^ NK cells into the TME. Delivering IL-2 or IL-15 expands NK cells, and delivering CD16 and 4-1BB agonists activates NK cells. Both methods can greatly improve sensitivity to RT. Another strategy is to increase the expression of TRAILs on NK cells or soluble TRAILs (sTRAILs), which leads to apoptosis of cancer cells. CAR-NK cells, with the advantage of being safer than CAR-T cells, may be an excellent adjunct to RT. B cells in the TME can differentiate into not only plasma cells to produce antibodies but also Bregs to impair the function of NK cells and Teff when the STING pathway is activated by STING agonists. PD-1, programmed cell death protein-1; CTLA-4, cytotoxic T lymphocyte-associated antigen-4; TIM-3, T-cell immunoglobulin and mucin domain-containing protein 3; TRAIL, tumor necrosis factor-related apoptosis-inducing ligand; TIGIT, T cell immune receptor with Ig and ITIM domains. NK cell, natural killer cell

The RT+dual ICI regimen might not be sufficient to treat patients with cold tumors. Only in the context of CD40 agonist treatment can IFN-γ cause inflammatory monocytes to secrete matrix metalloproteinases capable of decomposing the extracellular matrix of PDAC. Rech et al. found that quadruple therapy with RT+αCD40 agonistic mAb+anti-PD1+anti-CTLA-4 (RCP4) reduced the prevalence of immunosuppressive γδT cells abundant in PDA and was associated with significantly enhanced outcomes. Almost 91% of RT-treated mice had complete regression of the irradiated tumor and decreased growth of the unirradiated tumor, which was not observed in mice treated with a triple regimen without a CD40 agonist.^189^ Wang et al. reported that γδ-T-Exos directly targeted radioresistant CD44^+/high^ cancer stem cells (CSCs) and eradicated nasopharyngeal carcinoma (NPC) tumor cells.^165^ Complete tumor regression was observed in a GBM mouse model because pretreatment with RT allowed CAR-T cells to rapidly extravasate from the vasculature and expand in the TME.^190^ In TNBC expressing EGFR, RT promoted the transendothelial migration of CAR-T cells by activating the NF-κB pathway to induce ICAM1 expression on TNBC cells and endothelial cells, providing a long-lasting antitumor effect when combined with EGFR-targeted CAR-T therapy^191^; these results indicate that the combination of CAR-T therapy and RT may overcome the lack of T cell infiltration in cold tumors^5,166^ (Fig. 4).

## Nature killer cells

Based on the expression of CD56 and CD16, NK cells can be divided into two major subtypes, CD56^bright^CD16^−^ NK cells and CD56^dim^CD16^+^ NK cells. CD56^bright^CD16^−^ NK cells can release abundant cytokines, such as IFN-γ and TNF-α, and chemoattractants, such as CCL5 and XCL1, to attract cDC1s to the TME.^192^ CD56^dim^CD16^+^ NK cells account for a large proportion of NK cells in the blood and directly kill cancer cells by secreting perforin and granzyme in the early stage of killing and by expressing FasL and TRAIL in the later stage to induce cancer cell apoptosis without the need for prior antigen priming.^193–195^

In a pancreatic cancer model, cellular senescence triggered by RT activated the NF-κB pathway. The NF-κB transcription factor RelA was essential for CXCL8 release from irradiated tumor cells, which caused CD56^dim^ rather than CD56^bright^ NK cells to migrate to the tumor.^196^ In addition, RT was also found to activate the mTOR pathway to reduce the expression of IκB-α, an NF-κB inhibitory protein, to promote CXCL8 release.^196^ However, the function of NK cells is limited. Both Colo829 and DU145 cells treated with either 3 × 8 Gy or a single dose of 16 Gy were resistant to NK cell cytotoxicity in vivo 72 h after RT because perforins released by NK cells failed to form functional pores in the membranes of irradiated cancer cells.^197^ Thus, combination therapies are necessary.

RT can upregulate both activating (i.e., NKG2D, NKG2C, NKp30, NKp40) and inhibitory (i.e., NKG2A) receptors.^198–200^ Rae-1 and ULBP, ligands of NKG2D on cancer cells, are upregulated by RT and histone deacetylase inhibitor (HDACi) therapy, which increased the cytotoxicity of NK cells against cancer cells.^201–204^ Therefore, RT can cause CSCs to become susceptible to NK cell-mediated cytotoxicity, and the combination of RT and HDACi therapy further amplifies the upregulation of rae-1.^203,205^ However, blocking the NKG2A inhibitory receptor did not improve either the cytotoxicity of NK cells or the effects of RT in both MOC2 and B16F10 models. Only a triplet regimen comprising RT+NKG2A blockade+anti-PD−1 therapy improved the survival rate of B16F10 mice, although the improvement was related to T cells rather than NK cells.^199,202^ Treating irradiated B16F10 tumors with α-CD16/α-4-1BB NPs, designed to simultaneously activate two costimulatory receptors, was shown to lead to greater tumor volume reduction than treatment with a mixture of α-CD16 NPs and α-4-1BB NPs.^201^ The combination of RT and an ATR inhibitor (ATRi) increased NK cell activation and TIGIT expression in NK cells in HNSCC patients. Combination blockade of TIGIT improved the tumor growth control induced by RT+ARTi therapy in the MOC2 model and thus can be considered a novel strategy to improve therapeutic effects.^206^

RT can also upregulate both PD-1 in NK cells and PD-L1 in NPC cells via the NF-κB pathway to enable tumor cells to escape the immune system. Administrating IFN-β is widely used as a maintenance therapy to upregulate membrane-bound TRAIL on NK cells and increase the release of soluble TRAIL (sTRAIL) by NK cells to induce apoptosis of target cells.^207^ Thus, blocking the PD-1/PD-L1 checkpoint can increase the cytotoxicity of IFNβ-activated NK cells to enhance the effect of RT.^208^

NK cell-based immunotherapy includes not only adoptive NK cell therapy by infusing expanded NK cells or modified NK cells (known as chimeric antigen receptor NK cells) into patients but also means to enhance NK cell activity in vivo via cytokine-based treatment, NK cell engagers and immune-checkpoint inhibition.^195^ Unfortunately, NK cell-based immunotherapy is not effective in solid tumors because the inhibitory TME of solid tumors features by hypoxia and fewer nutrients, and those NK cells cannot enter the tumor. However, RT may overcome this issue and enhance the therapeutic effect of traditional NK cell immunotherapy.^205^ The combination of RT and NK cell adoptive transfer has already proven effective in human TNBC xenograft tumor models, in which it was successfully reported to control both the primary tumor and metastases.^200^

NK cell expansion and enhancement of NK cell cytotoxic activity in vitro are important for adoptive NK cell therapy. The traditional method is to use the chemokines IL-12, IL-15, and IL-18.^209^ In comparison with the traditional method, 24 h treatment with LDRT (optimal dose, 75 mGy) might be a more convenient way to expand NK cells and might be associated with the P38-MARK pathway.^210^ After stimulation by IL-2 or IL-15, CD56^bright^CD16^−^ NK cells can also obtain high cytotoxicity.^194^ The IL-2 receptor consists of CD25, CD122 and CD132, with the last two subunits also belonging to the IL-15 receptor. In cytokine-based therapy, subcutaneously delivering IL-15 with local RT given at 3 × 8 Gy had synergistic effects in both mammary carcinoma and colorectal carcinoma mouse models.^211^ However, CD25 is expressed on both NK cells and Tregs, and thus, IL-2 is rarely used instead of IL-15 to expand NK cells because IL-2 also activates Tregs.^199^ Blocking CD25 can deplete Tregs, leading to the accumulation of more IL-2, and activate NK cells by forcing IL-2 to bind CD122 to produce FLT3L to activate DCs.^199,205^ The triple regimen of RT, anti-CD25 therapy and an anti-CD137 agonistic antibody was found to be successful in HNSCC models. Moreover, FLT3L treatment can reverse the depletion of NK cells to enhance this therapy.^199,212^

Phase I trials showed that chimeric antigen receptor NK cell (CAR-NK) therapies, such as CAR-NK-92, could be attractive and effective strategies because they were found to be safer than CAR-T cell therapy due to their lower associated risk of graft-versus-host disease (GVHD), cytokine release syndrome (CRS) and neurotoxicity. Therefore, clinical trials on RT in combination with CAR-NK cell therapy might not be far off (Fig. 4).

## B cells

Similar to T cells, tumor-infiltrating B cells (TIL-Bs) can be divided into effector and regulatory B cells (Bregs).^213^ TIL-Bs are mainly present in the tertiary lymphoid structures (TLSs) and lymph-myeloid aggregates (LMAs) of tumors.^213^ TLSs are marked by a distinct germinal center surrounded by a mix of CD20^+^ B cells and CD3^+^ T cells. CD3^+^ T cells include both CD8^+^ and CD4^+^ T cells.^214^ Exhausted or dysfunctional CD8^+^ and CD4^+^ TILs express CXCL13 to attract B cells into TLSs by binding CXCR5. B cells are also regarded as APCs. They can process and present MHC I and MHC II to CD8^+^ and CD4^+^ T cells and promote T cell responses. Comparatively, Bregs suppress the activation of CD8^+^ T cells and NK cells by expressing IL-10, IL-35, and TGF-β^213^ via the STING/IRF3 pathway. Studies have found that in a mouse model of pancreatic cancer, the addition of IL-35-blocking antibodies to STING agonists effectively improved the effects of tumor-infiltrating NK cells, thereby achieving antitumor effects.^215^ B cells can also differentiate into plasma cells in TLSs and LMAs, and patients with many B cells and TLSs and a high plasma cell signature had a longer OS and better prognosis when treated with ICIs.^214,216–218^

Following RT at a dose of 11.7 Gy, TLSs in the lungs of KP mice demonstrated a fivefold reduction in size, and 14 days after RT, the TLS had returned to a normalized state. However, no significant change was observed in the number of B cells two hours after RT. Subsequently, 36 h after RT, the proportion of B cells was decreased by 35%, while the proportion of CD4^+^ T cells, which primarily consisted of Tregs, was decreased by 30%. As the size of TLSs returned to normal, the above immune cells also normalized.^219^ The same phenomenon was observed in PDAC patients undergoing RT (25 Gy in 5 fractions).^220^ RT at doses of 12–18 Gy not only promoted the activation of B cells in lymph nodes but also enhanced the generation of plasma cells and antibodies in a mouse model of human papillomavirus-associated head and neck squamous cell carcinoma (HPV-associated HNSCC). Concurrently, the number of Bregs was found to increase.^217^ Therefore, further investigations are warranted to elucidate the implications of these changes in tumor-infiltrating B cells.

There is a relative paucity of research on B cells compared to T cells, particularly studies examining the effects of different doses and regimens of RT on B cell populations within tumors. Though SM16 enhances the effects of RT by influencing TAMs and TANs, it is not known whether this combination is involved in B cell changes by inhibiting the function of Bregs.^111^ Strategies for activating B cells, such as eliminating Bregs and adoptive transfer of B cells, are still being developed. Analysis of tumor-specific antibodies in the serum of cancer patients can provide insight into the activity of plasma cells, but it cannot definitively establish whether these antibodies are produced by plasma cells located within the tumor or those located in lymph nodes. Therefore, determining the number and function of plasma cells in tumors following RT remains challenging.^221^ Although B cells are not as effective as T cells in tumor immunity, using tumor-specific antibodies produced by plasma cells may greatly increase the accuracy and efficacy of combination therapies. (Fig. 4)

## Conclusion

In summary, the immune cell populations present within the TME are diverse and complex and cannot be dichotomized as solely immunosuppressive or solely immunostimulatory because the phenotypic changes exhibited by immune cells in the TME are highly variable and depend on the unique features of the specific TME being considered. With the development of single-cell sequencing technology, an increasing number of discoveries related to different subpopulations of the same cells are being made, but subtle changes in the numbers and functions of these subpopulations after RT are still difficult to accurately monitor. The same tumors but different individuals, the same tumor in the same specie but different subtypes, and the same subtype but different stages are all factors influencing the proportion and functions of immune cells in the TME. Against the backdrop of these variables, target volumes, different single doses and fractionation schedules can affect the efficacy and prognosis of combination therapy and lead to differences in outcomes.

The purposes of combination therapy can be summarized as follows. First, combination therapy aims to amplify the beneficial effects of RT, such as promoting the infiltration and activation of immune-stimulating cells caused by RT. Second, combination therapy aims to reduce the negative effects caused by RT, for example, by depleting immunosuppressive cells and reversing transformation into an immunosuppressive or exhausted phenotype. In particular, triple or quadruple therapies can maximize the benefits and perpetuate the effects of combination therapy. It is also important to carefully determine the optimal sequence, timing and route of medications. In this regard, nanotechnology is a safer, more effective, and more accurate delivery system for drugs or exosome cargo and can even produce better effects. However, there are still some challenges that have not been resolved. Although we have achieved impressive results in mouse models, due to species differences, it remains difficult to accurately determine the radiation dose, as what is applied in mouse models cannot be directly translated to humans. Currently, over a dozen clinical trials are evaluating the therapeutic effects of drugs with different targets in combination with RT (Table 2). However, translating experimental findings from mouse models to humans remains challenging, and addressing potential side effects, such as CRS and other immune-related adverse events, is critical. Ultimately, resolving these challenges requires further research and validation of speculative hypotheses.

**Table 2:** Representative trials using combination of radiotherapy and immunotherapy

Clinical trials.gov identifier	Radiation	Drug	Type of cancer	Phase	Targets
NCT04786093	SABR	Durvalumab	NSCLC	Phase 2	PD-L1/B7-1
NCT04889066	SABR	Durvalumab	Brain Metastases From NSCLC	Phase 2	PD-L1/B7-1
NCT02180698	RT	GLA-SE	Stage III/IV Adult Soft Tissue Sarcoma	Phase 1	TLR4
NCT02556463	Palliative RT	MEDI9197	Solid Tumors	Phase 1	TLR7/8
NCT02927964	RT	SD-101	Follicular Lymphoma	Phase 1, Phase 2	TLR9
NCT03410901	RT	SD-101	Low-Grade B-Cell Non-Hodgkin Lymphomas	Phase 1	TLR9
NCT04050085	RT	SD-101	Refractory Metastatic Pancreatic Cancer	Phase 1	TLR9
NCT00185965	RT	CPG 7909	Non-Hodgkin Lymphoma, Mycosis fungoides	Phase 1, Phase 2	TLR9
NCT04995536	RT	CpG-STAT3 siRNA CAS3/SS3	Relapsed/Refractory B-Cell Non-Hodgkin Lymphoma	Phase 1	TLR9
NCT02254772	RT	SD-101	Low-Grade B-Cell Lymphomas	Phase 1, Phase 2	TLR9
NCT03007732	SBRT	SD-101	Hormone-Naïve Oligometastatic Prostate Cancer	Phase 2	TLR9
NCT03507699	Liver RT	CMP-001	Liver Metastases in Colorectal Carcinoma	Phase 1	TLR9
NCT01976585	RT	Poly-ICLC	Low-Grade B-cell Lymphoma	Phase 1, Phase 2	TLR3
NCT03610711	SBRT	Relatlimab	Gastroesophageal Cancer	Phase 1, Phase 2	LAG3
NCT03423628	RT	AZD1390	Brain Cancer	Phase 1	ATM
NCT05002140	Palliative RT	XRD-0394	Metastatic, Locally Advanced, or Recurrent Cancer	Phase 1	ATM
NCT02223923	Palliative RT	AZD6738	Refractory Solid Tumor	Phase 1	ATR
NCT04576091	SBRT	BAY 1895344	Recurrent Head and Neck Squamous Cell Carcinoma	Phase 1	ATR
NCT03022409	RT	AZD6738	Head and Neck Squamous Cell Carcinoma	Phase 1	ATR
NCT04049669	Palliative Full-dose RT	Indoximod	Glioblastoma, Medulloblastoma, Ependymoma, Diffuse Intrinsic Pontine Glioma	Phase 2	IDO
NCT04047706	RT	BMS-986205	Glioblastoma	Phase 1	IDO
NCT04192981	Whole-Brain RT	GDC-0084	PIK3CA-Mutated Solid Tumor Brain Metastases or Leptomeningeal Metastases	Phase 1	PI3K
NCT00704080	RT	XL765 (SAR245409)	Mixed Gliomas, Malignant Gliomas, Glioblastoma Multiforme	Phase 1	PI3K
NCT02128724	Palliative TRT	BKM120 (Buparlisib)	Carcinoma, Non-Small-Cell Lung	Phase 1	PI3K
NCT03696355	RT	GDC-0084(Paxalisib)	Brain and Central Nervous System Tumors	Phase 1	PI3K
NCT02537223	IMRT	BYL719	Squamous Cell Carcinoma of Head and Neck	Phase 1	PI3Kα
NCT05009992	RT	GDC-0084(Paxalisib)	Diffuse Midline Gliomas	Phase 2	PI3K
NCT02113878	IMRT	BKM120 (Buparlisib)	Advanced Squamous Cell Cancer of Head and Neck	Phase 1	PI3K
NCT02581787	SBRT	Fresolimumab	NSCLC	Phase 1, Phase 2	TGF-β
NCT04560244	Hypo-Fractionation RT	SHR1701	NSCLC	Phase 2	PD-L1/ TGF-β RII
NCT00051467	RT	TNFerade™	Pancreatic Cancer	Phase 3	TNF
NCT05433597	RT	Tianenfu	Nasopharyngeal Carcinoma	Phase 2, Phase 3	TNF
NCT04491084	SBRT	CDX-1140	NSCLC	Phase 1, Phase 2	CD40
NCT0114675	TRT	AZD6244 (Selumentinib)	NSCLC	Phase 1	MEK
NCT0174064	RT	GSK1120212 (Trametinib)	KRAS, BRAF, and NRAS-MUTANT Rectal Cancers	Phase 1	MEK
NCT03919071	RT	Dabrafenib Mesylate (GSK2118436B)	High-Grade Glioma	Phase 2	BRAF/ MEK
NCT02015117	Whole-Brain RT	GSK1120212 (Trametinib)	Brain Metastatic Malignant Neoplasm	Phase 1	MEK
NCT03975231	IMRT	GSK1120212 (Trametinib)	BRAF Mutated Anaplastic Thyroid Cancer	Phase 1	MEK
NCT05292417	Hypo-Fractionation RT	GM-CSF	Colorectal Neoplasms	Phase 2	GM-CSF
NCT04892498	Hypo-Fractionation RT	GM-CSF,IL-2	Advanced Refractory Solid Tumors	Phase 2	GM-CSF, IL-2
NCT05407649	RT	GM-CSF	Thymoma	Phase 2	GM-CSF
NCT04106180	SBRT	GM-CSF	NSCLC	Phase 2	GM-CSF
NCT02648282	SBRT	GVAX	Pancreatic Cancer	Phase 2	GM-CSF
NCT00021333	RT	Filgrastim	Head and Neck Cancer, Lung Cancer	Phase 2	G-CSF
NCT00113230	RT	Bevacizumab	Rectal Cancer	Phase 2	VEGF
NCT00557492	EBRT	Avastin	Pancreatic Cancer	Phase 2	VEGF
NCT04785287	SBRT	BMS-986218	Metastastic Solid Malignancies	Phase 1, Phase 2	CTLA4
NCT03601455	External beam RT	Tremelimumab	Bladder Cancer	Phase 2	CTLA4
NCT03507699	Liver RT	Ipilimumab	Liver Metastatic Colorectal Cancer	Phase 1	CTLA4
NCT05484024	Short-course RT	Sintilimab	Malignant Rectal Neoplasms	Phase 2, Phase 3	PD-1
NCT03898895	SBRT, IMRT	Camrelizumab	Biliary Tract Cancer	Phase 2	PD-1
NCT05554276	Radical RT	Camrelizumab	Cervical Cancer	Phase 2	PD-1
NCT04936841	Palliative RT	Pembrolizumab	Head and Neck Cancer	Phase 2	PD-1
NCT03187314	RT	SHR-1210	Esophageal Cancer	Phase 2	PD-1
NCT03984357	IMRT	Nivolumab	Nasopharyngeal Carcinoma	Phase 2	PD-1
NCT04977375	SBRT	Pembrolizumab	Glioblastoma Multiforme	Phase 1, Phase 2	PD-1
NCT05659186	RT	Tislelizumab	Thyroid Cancer	Phase 2	PD-1
NCT04748419	Hypo-Fractionation RT	Durvalumab	NSCLC	Phase 1, Phase 2	PD-L1
NCT03955978	Brachytherapy	TSR-042	Endometrial Cancer	Phase 1	PD-1
NCT03044626	RT	Nivolumab	NSCLC	Phase 2	PD-1
NCT02608385	SBRT	Pembrolizumab	Solid Tumor	Phase 1	PD-1
NCT03262454	Hypo-Fractionation RT	Atezolizumab	SCLC	Phase 2	PD-L1
NCT03050554	SBRT	Avelumab	NSCLC	Phase 1, Phase 2	PD-L1
NCT02992912	SABR	Atezolizumab	Metastatic Tumors	Phase 2	PD-L1
NCT04690855	RT	Atezolizumab	Triple Negative Breast Cancer	Phase 2	PD-L1
NCT03051906	IMRT	Durvalumab	Head and Neck Neoplasms	Phase 1, Phase 2	PD-L1
NCT04992780	Hypo-Fractionation RT, Standard-Fractionation RT	Durvalumab	NSCLC	Phase 2	PD-L1

*RT* radiation therapy, *SABR* stereotactic ablative radiotherapy, *SBRT* stereotactic body radiation therapy, *IMRT* intensity-modulated radiation therapy, *EBRT* external beam radiation therapy, *NSCLC* non-small cell lung cancer, *SCLC* small cell lung cancer
